# Supplementary material for: Untreated Vestibular Schwannoma: Analysis of the Determinants of Growth
Source: Cancers (Basel). 2024 Nov 4;16(21):3718. doi: 10.3390/cancers16213718 (PMC11545831; doi:10.3390/cancers16213718)
Supplement: Supplementary file 1 [file cancers-16-03718-s001.zip › cancers-3264963-supplementary.pdf]

Reference	Selection				comparability	Outcome			Score
	Representative ness of the ex-	Selection of the nonexp-	Ascertainm en of inter-	Demonstration that outc- ome of int-erest was not	.Comparability of cohorts on the basis of	Assessme- nt of	Was follow-up long enough for	Adequacy of follow up to	
MarcDiensthuber 2005	★	★	★	☆	★☆	★	★	☆	6
DAVID FLINT,2005	★	★	★	☆	★★	★	★	☆	7
Amit Herwadker,2005	★	★	★	☆	★☆	★	★	★	7
Sven-Eric S,2006	★	★	★	☆	★★	★	★	★	8
C. Arturo Solares,2008	★	★	★	☆	★★	★	★	☆	7
Wissame El B,2009	★	★	★	☆	★★	★	★	☆	7
K. WHITEHOUSE,2010	★	★	★	☆	★☆	★	★	★	7
RSURYANARAYANAN,2010	★	★	★	☆	★★	★	★	★	8
Yuri Agrawal,2010	★	★	★	☆	★★	★	★	☆	7
Ferdinand C. A. T,2011	★	★	★	☆	★★	★	☆	☆	6
Mark Hughes,2011	★	★	★	☆	★★	★	★	★	8
Cathrine N Breivik,2011	★	★	★	☆	★☆	★	★	★	7
David A. Moffat,2012	★	★	★	☆	★☆	★	★	☆	6
Jong Dae Lee,2014	☆	★	★	☆	★☆	★	★	☆	5
Ricardo José G.,2014	★	★	★	☆	★☆	★	★	☆	6
Daniel Jethanamest,2015	★	★	★	☆	★★	★	★	☆	7
Yosuke Tomita,2015	★	★	★	☆	★★	★	★	★	8

Wolbers, J.G,2016	★	★	★	☆	★★	★	★	★	8
Charles R.J,2016	★	★	★	☆	★☆	★	★	☆	6
Prasad, S. C.,2017	★	★	★	☆	★☆	★	★	☆	6
Jacob B. Hunter,2017	★	★	★	☆	★☆	★	★	☆	6
Katherine A. Lees,2018	★	★	★	☆	★☆	★	★	☆	6
Maarten Kleijwegt,2019	★	★	★	☆	★★	★	★	☆	7
Siska D’ Haese,2019	★	★	★	☆	★★	★	★	☆	7
Daniele Borsetto,2019	★	★	★	☆	★★	★	★	☆	7
Mantegh Sethi,2020	★	★	★	☆	★★	★	★	☆	7
Zane Schnurman,2020	★	★	★	☆	★☆	★	★	☆	6
Martin Reznitsky,2020	★	★	★	☆	★☆	★	★	★	7
Kathrin Skorpa，2020	★	★	★	☆	★☆	★	★	★	7
Maxime Fieux,2020	★	★	★	☆	★★	★	★	★	8
John P. Marinelli,2021	★	★	★	☆	★☆	★	★	★	7
John P. Marinelli,2021	★	★	★	☆	★☆	★	★	☆	6
Jong Sei Kim,2021	★	★	★	☆	★★	★	★	★	8
Yoshinori Higuchi,2021	★	★	★	☆	★★	★	★	★	8
Mayke A. Hentschel,2021	★	★	★	☆	★☆	★	★	★	7
A Dardis,2021	★	★	★	☆	★☆	★	★	☆	6
Hiroyuki Yamada,2022	★	★	★	☆	★★	★	★	☆	7
Takashi Itoyama,2022	★	★	★	☆	★☆	★	★	☆	6
Kazutake Yagi,2023	★	★	★	☆	★★	★	★	★	8
Truong, L. F.,2023	★	★	★	☆	★☆	★	★	☆	6
John P. Marinelli,2023	★	★	★	☆	★☆	★	★	☆	6

Legend

Table S1: This table presents the NOS scale scores of 41 studies, which assess the risk of bias by assessing the quality of cohort studies in three aspects: selectivity, comparability, and outcome.

The **black stars** represent the scores obtained, while the **white stars** represent the scores not obtained. The total score is 9 points, with a score greater than 7 indicating high-quality research
